# Important Determinants for Fucoidan Bioactivity: A Critical Review of Structure-Function Relations and Extraction Methods for Fucose-Containing Sulfated Polysaccharides from Brown Seaweeds

**DOI:** 10.3390/md9102106

**Published:** 2011-10-24

**Authors:** Marcel Tutor Ale, Jørn D. Mikkelsen, Anne S. Meyer

**Affiliations:** Center for Bioprocess Engineering, Department of Chemical and Biochemical Engineering, Technical University of Denmark (DTU), Søltofts Plads, Building 229, DK-2800 Kgs. Lyngby, Denmark; E-Mails: mta@kt.dtu.dk (M.T.A.); jdm@kt.dtu.dk (J.D.M.)

**Keywords:** fucoidan, antitumor, anticoagulant, extraction, sulfated polysaccharides

## Abstract

Seaweeds—or marine macroalgae—notably brown seaweeds in the class Phaeophyceae, contain fucoidan. Fucoidan designates a group of certain fucose-containing sulfated polysaccharides (FCSPs) that have a backbone built of (1→3)-linked α-l-fucopyranosyl or of alternating (1→3)- and (1→4)-linked α-l-fucopyranosyl residues, but also include sulfated galactofucans with backbones built of (1→6)-β-d-galacto- and/or (1→2)-β-d-mannopyranosyl units with fucose or fuco-oligosaccharide branching, and/or glucuronic acid, xylose or glucose substitutions. These FCSPs offer several potentially beneficial bioactive functions for humans. The bioactive properties may vary depending on the source of seaweed, the compositional and structural traits, the content (charge density), distribution, and bonding of the sulfate substitutions, and the purity of the FCSP product. The preservation of the structural integrity of the FCSP molecules essentially depends on the extraction methodology which has a crucial, but partly overlooked, significance for obtaining the relevant structural features required for specific biological activities and for elucidating structure-function relations. The aim of this review is to provide information on the most recent developments in the chemistry of fucoidan/FCSPs emphasizing the significance of different extraction techniques for the structural composition and biological activity with particular focus on sulfate groups.

## 1. Introduction

Fucoidan is a term used for a class of sulfated, fucose rich, polysaccharides found in the fibrillar cell walls and intercellular spaces of brown seaweeds of the class Phaeophyceae. These fucose-containing sulfated polysaccharides (FCSPs) principally consist of a backbone of (1→3)- and (1→4)-linked α-l-fucopyranose residues, that may be organized in stretches of (1→3)-α-fucan or of alternating α(1→3)- and α(1→4)-bonded l-fucopyranose residues. The l-fucopyranose residues may be substituted with sulfate (SO_3_^−^) on the C-2 or C-4 (rarely on C-3), with single l-fucosyl residues and/or with short fucoside (fuco-oligosaccharide) side chains. If present, the fucoside side chains are usually O-4 linked to the α-l-fucopyranose backbone residues. However, as FCSPs structures of more brown seaweeds are being elucidated, as discussed further in the present review, it appears that FCSPs cover a broader range of complex polysaccharides than only those having fucan backbones. Apart from variations in the sulfate content and substitutions, also the monosaccharide composition of FCSPs varies among different species of brown seaweeds. Hence, in addition to fucose, different types of FCSPs may also contain galactose, mannose, xylose, glucose and/or glucuronic acid—usually in minor amounts [1].

According to the ISI Web of Knowledge (Thomson Reuters) the number of published articles with the topic assigned as “fucoidan” has increased significantly since fucoidan, or “fucoidin” as it was first called, was first isolated from brown algae in 1913 [2]; in particular, a profound increase in the number of papers has taken place during the last 5–10 years. By now, the published papers related to fucoidan hit approximately 1800 (August 2011, Figure 1). The recent interest has mainly focused on the potentially beneficial biological activities of fucoidan and FCSPs in humans including antitumor, immunomodulatory, anti-inflammatory, antiviral, antithrombotic, anticoagulant, and antioxidant effects as well as specific activities against kidney, liver and urinary system disorders.

While the development of research efforts involving FCSPs and their potential applications are advancing, the understanding of the mechanisms and the particular structural features of the FCSPs being responsible for the various biological activities is still incomplete. Seaweeds, including various brown seaweeds such as *Undaria* and *Laminaria* spp., are part of the food culture in Asia, notably in Japan, the Philippines, and Korea, and seaweed extracts have also been used as a remedy in traditional medicine. However, no standardized FCSPs extraction or purification protocols exist, and no specific pharmaceutical, dermatological, or nutraceutical applications have as yet been officially approved for these polysaccharides or their lower molar mass oligosaccharide derivatives.

It is our proposition that more focus should be directed to the extraction and purification processes in order to obtain consistent protocols that account for the biodiversity of FCSPs from different seaweeds and to retain the structural features of significance for the specific bioactivity properties of FCSPs. The development and use of such consistent extraction procedures would also help in achieving a better understanding of structure-function relationships of FCSPs. The aim of this review is to bring attention to the detailed structural features of FCSPs in relation to their marine algal sources and the extraction methodology, and to highlight recent knowledge concerning the structural determinants for FCSPs bioactivity.

## 2. Historic Overview: FCSPs Extraction Procedures and Chemical Analyses

Extraction using dilute acetic acid followed by purification was used by Kylin already in 1913 to isolate “fucoidin”, subsequently referred to as fucoidan, from various species of *Laminaria* and *Fucus* [2]. Already in this first seminal report, Kylin reported that fucoidan extracted in this way mainly contained fucose, but also observed that the fucose occurred together with mannitol, alginic acid and laminarin [2] (Table 1); we now know that this interpretation was a result of co-extraction of these latter contaminants with the fucoidan. Two years later, Kylin reported that fucoidan isolated from *Laminaria digitata* contained methylpentose, interpreted as l-fucose, as well as some other pentoses [3]. A parallel report was published that same year by Hoagland and Lieb (1915) [4] who isolated a water-soluble polysaccharide from *Macrocystis pyrifera* that was closely related to if not identical with “fucoidan”, and shown to contain l-fucose as well as relatively high levels of calcium and sulfate. They employed a Na_2_CO_3_ soaking step and addition of hydrochloric acid which is why they also—if not mainly—isolated alginic acid (or alginate) during the extraction (Table 1). The rationale behind the extraction of alginate from the seaweed with Na_2_CO_3_ soaking is to convert all the alginate salts, typically calcium and magnesium alginate, to the sodium salt. Please note that the terminology used is confusing; alginic acid or alginate does not designate one particular monosaccharide or one type of homo-polysaccharide. With the advances in chemical analyses it is now known that alginic acid or alginate is comprised of guluronic and mannuronic acids; which are C-5 epimers. Structurally, alginic acid, or alginate, is a linear hydrocolloid polymer that consists of blocks of (1→4) linked β-d-mannuronate and α-l-guluronate residues. The detailed structure of alginate may have mannuronate and guluronate in homopolymeric blocks of consecutive mannuronate residues (M-blocks), in consecutive guluronate residues (G-blocks), or in structural units of alternating mannuronate and α-l-guluronate residues (MG-blocks). Both mannuronic acid and α-l-guluronic acids are uronic acids, and they have in the past been analytically determined as uronic acids. However, uronic acids also include several other structures, e.g., glucuronic and galacturonic acids. In this review, we will address the significance of the compositional and structural differences, but also attempt to introduce a consistent terminology by grouping these compounds as uronic acids, especially as homopolymers do not belong to the group of FCSPs. Hoagland and Lieb (1915) [4] did not compare their extraction with Na_2_CO_3_ soaking to one without, but their report is nevertheless the first example of how the extraction procedure may influence the purity and, in turn, the analyzed composition of the extracted FCSPs (see Table 1).

The early FCSPs extraction procedures were based on using a dilute acid treatment, with either acetic or hydrochloric acid used as a first “extraction” step with the purpose of hydrolyzing the non-FCSP polysaccharides (Table 1). However, the extraction and purification methodologies employed in different studies to isolate fucoidan/FCSPs from brown seaweed biomass have been modified to different extents since the first reports from 1913 and 1915. Bird and Haas (1931) [5], for example, used soaking of the brown alga biomass in water and precipitation of crude sulfate from the extract with ethanol to obtain fucoidan from *L. digitata* (Table 1). The product of this extraction was also found to contain relatively high levels of ash. The high ash levels were presumably chiefly a result of the presence of calcium sulfate in the algal polysaccharides. For the compositional analysis, acid hydrolysis using H_2_SO_4_ was typically used. This acid hydrolysis step might by itself have contributed a substantial amount of sulfate. As a consequence, the use of H_2_SO_4_ clearly biased the interpretation of the compositional analysis. Hence, the fucoidan isolated by Bird and Haas (1931) [5] was designated as carbohydrate sulfate (*i.e.*, containing esterified sulfate) since the total sulfate content was approximately the double of that found in the ash. Uronic acid was also present in the FCSPs preparation extracted from *L. digitata* [5] (Table 1).

Nelson and Cretcher (1931) [6] extracted fucoidan from *Macrocystis pyrifera* by repeated, extended (48 h) extraction with dilute HCl followed by isolation of the FCSPs by ethanol precipitation, and revealed the presence of sulfate in the form of ester groups in the precipitated product. They also confirmed that fucose was the only sugar identified in the unhydrolyzed residue after acid hydrolysis, even though their product contained uronic acid, considered to be due to alginate contamination (Table 1). Later, Lunde *et al.* [7] prepared fucoidan by directly precipitating the droplets exuded from freshly gathered *L. digitata* fronds in ethanol (Table 1). After purification via precipitation of the product from boiling ethanol they obtained a FCSPs specimen that contained 33–37% methylpentose (interpreted correctly as fucose), and 26–30% ash in which the sulfate content was 17–19%, which made up approximately half of the total sulfate estimated in the polysaccharide (35–38%). They proposed a structural unit formula, (R-R′-O-SO_2_-OM)*_n_*, for fucoidan and suggested that R was fucose or another pentose sugar residue, R′ was unknown, and M was Na^+^, K^+^, (½)Ca^2+^ or (½)Mg^2+^ [7] (Table 1).

“In an attempt to reconcile some of the conflicting views on the nature of fucoidin” crude fucoidan extracts from *F. vesiculosus*, *F. spirales*, *Himanthalia lorea* and *Laminaria clustoni* were prepared by Percival and Ross (1950) [8]. Their methodology involved boiling of the seaweed biomass in neat boiling water for 24 h (hydrolysis treatment) followed by removal of alginates and protein by addition of lead acetate, then, after addition of barium hydroxide (presumably to precipitate alginate) the fucoidan was isolated as a crude lead hydroxide complex (Table 1). In order to isolate lead free fucoidan, the lead hydroxide complex was treated with dilute H_2_SO_4_ and fucoidan was then isolated after prolonged dialysis and filtration. The purest fucoidan specimen obtained was from *H. lorea*. This fucoidan isolated from *H. lorea* contained substantial amounts of fucose and sulfate; as well as small quantities of uronic acid, galactose and xylose. Metals and ash were also detected, and the ash was mainly made up of calcium sulfate [8]. Based on the data obtained the authors believed that the principal constituent of fucoidan was a polyfucose with one sulfate substitution on each fucose residue and that other constituents arose from adventitious impurities. A parallel paper was published in which it was proposed that the core structure of fucoidan from *F. vesiculosus* was built of 1→2 linked l-fucopyranose units, each carrying a sulfate group on C-4 [10]. As discussed later, the interpretation that the fucosyl units in the fucoidan were 1→2 linked turned out to be incorrect, as more advanced analyses have now verified that the backbone of fucoidan from *F. vesiculosus* consists of alternating α(1→3) and α(1→4) linkages [11].

In the pursuit to obtain extensive quantities, a laboratory-scale extraction of fucoidan was reported by Black *et al.* [9]. Interestingly, the extracted product was referred to as a “polyfucose ethereal sulphate occurring in the *Phaeophyceae*”. Their optimal fucoidan extraction procedure was as follows: One part by weight of dried ground seaweed and 10 parts by volume of 0.1 M hydrochloric acid at pH 2.0–2.5 contacted at 70 °C with constant stirring for 1 h. A single acid hydrolysis extraction treatment using this method recovered about 50% by weight (w/w) of the theoretical maximum of fucoidan (recovered yield measured as % fucose obtained as % of total fucose in the seaweed dry weight), whereas three rounds of the acid extraction recovered more than 80% of the fucose present; the triple acid hydrolysis treatment (0.1 M HCl, pH 2.0–2.5, 70 °C, 1 h × 3) was therefore selected as the optimal extraction method (Table 1). After the acid hydrolysis treatment the crude fucoidan was isolated by fractional precipitation with alcohol and further purified by precipitation after addition of formaldehyde [9]. By this procedure samples of crude fucoidan containing 30–36% fucose were obtained; for example, the fucoidan recovered from *F. vesiculosus* using the optimal extraction protocol was analyzed to contain (by weight) 44% fucose; 26% total sulfate, and 31% ash [9]. In terms of yields, calculated as fucose as % of total fucose, the results obtained for the four different algal species were: *Pelvetia canaliculata* 76%; *F. vesiculosus* 62%; *Ascophyllum nodusum* 54%, and *L. cloustoni* 20% [9]. The authors suggested that a more efficient extraction methodology, *i.e.*, extracting higher fucose yields, could be achieved by increasing the water/seaweed ratio, extraction time or number of extractions.

In a study about 20 years later, FCSPs from the brown seaweeds *Himanthalia lorea*, *Bifurcaria bifurcata* and *Padina pavonia* were extracted successively using dilute acid, followed by alkaline or neat water extraction, acid, and alkali in sequence [12] (Table 2). Prior to extraction, the seaweed fronds were treated with formaldehyde to polymerize phenolic constituents which might otherwise contaminate the extracted saccharides [12]. This extraction protocol produced a complex mixture of glucans, fucose-containing polysaccharides, and alginic acid which could be separated by fractional precipitation with ethanol, calcium salts (CaCl_2_) or by fractionation on resin columns. The FCSPs extracted in this fashion were reported to be heteropolysaccharides containing different levels of fucose, glucuronic acid, xylose, and esterified sulfate, together with traces of galactose [12]. The results also showed that the sulfate and uronic acid contents in the FCSPs separated from the aqueous calcium chloride and acid extracts varied significantly according to the seaweed species [12].

Another study was conducted on the purification of a sulfated heteropolysaccharide substance from *Sargassum linifolium* to elucidate its structural components [13]. The extraction was done using hydrochloric acid at pH 1.0 for 3 h at 80 °C (Table 2); the extract was then neutralized with aqueous sodium carbonate and precipitated with ethanol [13]. The resulting sulfated polysaccharide material, termed “sargassan”, was proposed to be built of glucuronic acid, mannose, and galactose residues with partially sulfated side-chains composed of galactose, xylose and fucose residues [13]. As discussed later, we now know that *Sargassum* spp. do indeed contain highly complex FCSPs structures built from this array of monosaccharides.

These early reports show that, with a few exceptions, treatment with dilute acid at ambient or slightly elevated temperature has been a preferred first step in extraction protocols for isolating fucoidan or FCSPs from different types of brown seaweeds (Tables 1 and 2). The use of different acids—or no acid at all—as well as the differences in extraction time and temperature during the extraction and further purification treatments have generated diverse compositional results and structural suggestions for fucoidan or FCSPs (Tables 1 and 2). The early reports almost unequivocally found that fucoidan mainly contained fucose and sulfate; nevertheless, the chemical composition of the most highly purified, but still crude fucoidan specimen from *Himanthalea lorea* indicated that the fucoidan of this seaweed species contained fucose, galactose, xylose, uronic acids as well as sulfate [8,14]. In the more recent reports, a pretreatment of the seaweed biomass prior to the real extraction treatment has been found to be advantageous to eliminate low molecular components (e.g., phenols); as already mentioned above, an early study used formaldehyde pretreatment [12]. However, more recent reports show that the pretreatment typically involves the use of a mixture of methanol, chloroform and water [22]. Removal of protein has also been considered. This can be facilitated via the use of proteolytic enzymes [28] (or by lead acetate treatment, as used by Percival and Roos in 1950 [8]). Another useful purification procedure has involved transformation of alginate in the residual biomass into insoluble calcium salts by treatment of the FCSPs specimen with aqueous calcium chloride [19,20].

In conclusion, the use of an array of different extraction and purification techniques appear to have contributed to the confusion that has prevailed about the nature and composition of fucoidan and FCSPs ever since fucoidan was first described by Kylin early in the 20th century [2]. As detailed in the following, we now know that the initial suggestions [29,30] that fucoidan was built of α(1→2) linked l-fucopyranosyl residues were wrong. Fucoidan is built of 1→3-linked α-l-fucopyranosyls or of alternating 1→3- and 1→4-linked α-l-fucopyranosyl residues that may be sulfate substituted. We also know,that “fucoidans” isolated from certain brown algae have completely different structures being composed of sulfated galactofucans with backbones of (1→6)-linked β-d-galacto- and/or (1→2)-β-d-manno-pyranosyl units with (1→3) and/or (1→4)-α-l-fucooligosaccharide branching. The available data thus show that the term “fucoidan” has been used for several different chemical structures and *vice versa* that fucoidan is a term that covers a diverse family of fucose-containing sulfated polysaccharides (Table 1). It is therefore more correct to use the term fucose-containing sulfated polysaccharides (FCSPs) rather than fucoidan as a collective term for these polysaccharides.

## 3. Taxonomic Comparison of Fucoidan or FCSPs Structure

### 3.1. Fucales

**Figure 2:**
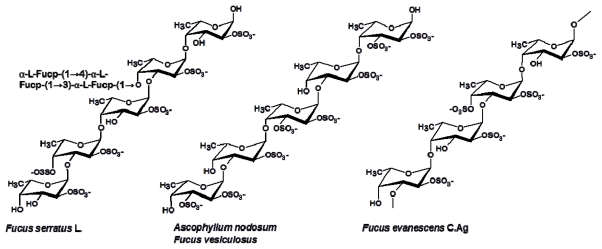
Typical structure of fucoidan (FCSPs) obtained from some brown seaweed species in the order of Fucales. The l-fucopyranose backbone of the fucoidan (FCSPs) extracted from *A. nodosum* and *F. vesiculosus* is connected by alternating α(1→3) and α(1→4) linkages [11]; The FCSPs from *F. evanescens* have a similar backbone built up with sulfate substituted at the 2- and 4-position of the fucose residues [20] (only sulfate substitutions on C-2 of fucose are shown in the Figure). Acetate substitutions may also be found at the C-4-position of 3-linked fucose and at C3 of 4-linked fucose units [22] (acetate substitutions not shown in the figure). For *F. serratus* L. a possible fucoside side chain at C-4 is also shown.

In 1993 a revised structure of the polysaccharide backbone of the main FCSP product from *F. vesiculosus* was presented as α(1→3) linked instead of as α(1→2) linked [31]; it was also reported that fucose was attached to the backbone fucan polymer to form branching points, typically one for every 2–3 fucose residues within the chain, still with sulfate groups at position C-4 on the fucose units [31]. However, detailed analysis of the methyl derivatives obtained from partially desulfated *F. vesiculosus* polysaccharides revealed the presence of 2,4-di-*O*-methylfucose as well as 2,3-di-*O*-methylfucose which indicated the presence of both α(1→3) and α(1→4) linked fucose residues [32] (Figure 2). A similar structure was also determined for a FCSPs-derived oligosaccharide of about 8–14 monosaccharide units extracted from *Ascophyllum nodosum* (Fucales) [11] (Figure 2). More recently, several studies—using highly advanced analytical methods—have documented that fucoidan from brown seaweed in the order of Fucales such as *F. evanescens* and *F. serratus* do indeed contain large proportions of both α(1→3) and α(1→4) glycosidic bonds [20–22] (Figure 2). Structural analysis of a depolymerized low molecular weight fraction of fucoidan from *F. evanescens* by MALDI-TOF and tandem ESI mass spectrometry has moreover shown that this fraction contains oligosaccharides with and without sulfate substitutions and that it mainly consists of α(1→3)-linked fucose residues being esterified with sulfate at C-2 [33]. This more detailed analysis has also revealed the presence of minor components of mixed monosulfated fucooligosaccharides containing both 2-*O*- and 4-*O*-sulfated (1→4) bonded xylose and galactose residues: Xyl-(1→4)-Fuc, Gal-(1→4)-Fuc, Gal-(1→4)-Gal-(1→4)-Fuc, Gal-(1→4)-Gal [24]. Glucuronic acid (GlcA) was also detected as being a part of the non-sulfated fucooligosaccharides: Fuc-(1→3)-GlcA, Fuc-(1→4)-Fuc-(1→3)-GlcA, Fuc-(1→3)-Fuc-(1→3)-GlcA respectively [33].

Brown seaweed species in the order of Fucales have also been reported to contain very complex FCSPs structures having fucose and galactose in comparable amounts; these structures are generally referred to as sulfated galactofucans and are predominantly found among *Sargassum* species [18,34,35]. These sulfated galactofucans are mainly built of (1→6)-β-d-galactose and/or (1→2)-β-d-mannose units with branching points formed by (1→3) and/or (1→4)-α-l-fucose, (1→4)-α-d-glucuronic acid, terminal β-d-xylose and sometimes (1→4)-α-d-glucose [18]. Early studies also reported the existence of fucoglucuronans having a backbone of glucuronic acid, mannose and galactose residues with side chains of neutral and partially sulfated residues of galactose, xylose and fucose; notably present in *Sargassum linifolium* [13]. More recently, the FCSPs of this type extracted from the brown seaweed *Sargassum stenophyllum* (Fucales) were grouped into two different types: type I was found to contain a relatively high percentage of α-d-glucuronic acid and relatively few sulfate groups, while type II contained relatively small amounts of α-d-glucuronic acid and a high percentage of sulfate [18]. The type I polysaccharides were composed of a linear backbone formed mainly by (1→6)-β-d-galactose and/or (1→2)-β-d-mannose with branching chains formed by (1→3) and/or (1→4)-α-l-fucose, (1→4)-α-d-glucuronic acid, while in the type II polysaccharides the backbone was mainly built of short galactan chains [18].

**Figure 3:**
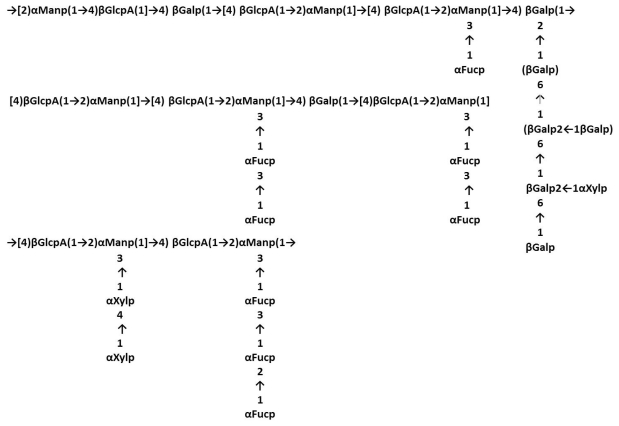
Suggested structures of the FCSPs (fucoidan) from *H. fusiforme* [23] also known as *Sargassum fusiforme* (Fucales); sulfate substitutions not shown. The structures also represent typical FCSPs structures from other *Sargassum* spp. [18].

Corresponding structures were observed in FCSPs fractions from *Hizikia fusiforme* a.k.a. *Sargassum fusiforme* (Fucales). These structures were separated by ion exchange chromatography after the FCSPs had been obtained via hot aqueous extraction, followed by ethanol and CaCl_2_ precipitation (Table 2). These chromatographically purified fractions predominantly contained fucose, mannose, galactose, uronic acid and sulfate [23] (Figure 3). In accordance with the findings of Duarte *et al.* [18] the structural analysis of one of the main fractions purified by ion exchange chromatography indicated that the sulfate groups might be found in any position on the galactose/mannose backbone or on the fucose units (Figure 3). The sulfate groups in the FCSPs in this fraction, which had been isolated after 3 rounds of extraction in hot water (70 °C), then ethanol and CaCl_2_ precipitation prior to chromatography (Table 2), were mainly found at C-6 of [→2,3)-Man-(1→], at C-4 and C6 of [→2)-Man-(1→] and at C-3 of [→6)-Gal-(1→] [23]. On the fucose, the sulfate groups were substituted at C-2, C-3 or C-4, while some fucose residues had two sulfate groups [23]. The core of these *S. fusiforme* FCSPs was mainly composed of alternating units of [→2)-α-d-Man-(1→] and [→4)-β-d-GlcA-(1→], with a minor portion of [→4)-β-d-Gal-(1→] units, and the branching points were at C-3 of [→2)-Man-(1→], C-2 of [→4)-Gal-(1→] and C-2 of [→6)-Gal-(1→], respectively [23] (Figure 3).

### 3.2. Laminariales and Other Brown Seaweed

Various structures of FCSPs from brown seaweeds of the order of Laminariales have also been reported [36]. The available data indicate that the FCSPs derived from this seaweed order contain small amounts of other monosaccharides besides fucose. Interestingly, polysaccharides containing significant amounts of fucose and galactose and which seem to be compositionally and structurally similar to the fucoidan from Fucales brown seaweeds appear to be prevalent [26,35]. Structural analysis was conducted on the FCSPs from the sporophyll *Undaria pinnatifida* (Laminariales) and it was found that the FCSPs had a high fucose/galactose ratio, high uronic acid, and low sulfate content. The most abundant fucopyranosyl units were substituted at the 3-; 2,3-; or 2,3,4-positions whereas fucose residues with substitutions at the 3,4- or 4-positions were less abundant [26]. The galactopyranosyl units were predominantly substituted at the 3- or at both the 3,4-positions [26].

**Figure 4:**
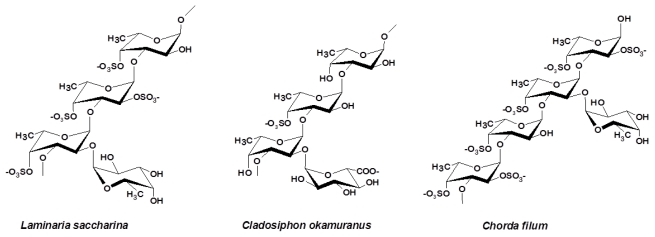
Structural motifs of FCSPs (fucoidan) from some brown seaweed species of the order Laminariales and Chordariales. FCSPs of *Chorda filum* and *Laminaria saccharina* consist of a poly-α-(1→3)-fucopyranoside backbone with sulfate mainly at C-4 and sometimes at the C-2 position; some of the backbone fucose residues may be acetylated at C-2 (not shown) [24,25]. *Cladosiphon okamuranus* derived FCSPs also consist of a backbone of α(1→3)-linked-l-fucopyranose residues with sulfate substitutions at C-4 and/or with α(1→2)-linked single α-l-fucopyranosyl substitutions and vicinal glucuronic acid substitutions. Some of the side chain fucose residues may be O-acetylated (not shown) [14].

FCSPs isolated from *Chorda filum* (Laminariales) have been shown to consist of a poly-α(1→3)-fucopyranose backbone with a high degree of branching mainly as α(1→2)-linked single α-l-fucopyranosyl residues (Figure 4) [24,25]; the fucopyranosyl residues were found to be sulfated mainly at C-4 and sometimes at the C-2 position, whereas some of the α(1→3)-linked fucose residues were shown by NMR to be C-2 acetylated [25]. A similar structure has been reported by Usov *et al.* [24] for the FCSPs isolated from *L. saccharina* (Laminariales) which are mainly built of (1→3)-linked α-l-fucopyranose with sulfation at C-4 and sometimes at the C-2 position or with possible α-l-fucopyranosyl at C-2 (Figure 4). This FCSPs structure has also been found to be present in the body wall layer of the sea cucumber *Ludwigothurea grisea* (a marine invertebrate). The FCSPs of the sea cucumber body wall are essentially built of an α-(1→3)-fucopyranose backbone [37]. NMR analysis has indicated that 2,4-di-sulfo-l-fucopyranose and unsubstituted fucopyranose are present in equal proportions, and that 2-mono-sulfo-l-fucopyranose is present in twice that proportion [33]. The FCSPs from *Lessonia vadosa* (Laminariales) have also been studied by NMR spectroscopy and the data indicate that the polysaccharides are mainly composed of α(1→3)-bonded fucopyranose residues sulfated mainly at position C-4 and partially at position C-2 [38].

Other algal fucoidans whose structures contain the same α(1→3)-backbone of fucose residues have been found in *Analipus japonicas* (Ectocarpales), *Adenocystis utricularis* (Ectocarpales) and *Cladosiphon okamuranus* (Chordariales) [14,15,39], but the FCSPs from these brown algae also appear to contain other monosaccharides than fucose (Table 2). The detailed structural elucidation of the FCSPs from *C. okamuranus* (Chordariales) confirmed that this product was made up of a linear backbone of α(1→3)-fucopyranose units with a portion of the fucose residues carrying sulfate substitutions at C-4 but some of the fucose residues have also been found to be O-acetylated (Figure 4). The *C. okamuranus* FCSPs may also contain α-glucuronic acid substitutions at the C-2-positions of those backbone fucose residues that are not substituted by a sulfate group [14] (Figure 4).

Methylation analysis, desulfation and NMR spectroscopy of the FCSPs fractions from *Adenocystis utricularis* (Ectocarpales) showed that these FCSPs contained the same α-(1→3)-fucopyranose backbone as that found in *Chorda filum* and *Laminaria saccharina* FCSPs, and that the fucopyranosyl units were mostly sulfated at C-4, and branched at C-2 with non-sulfated fucopyranosyl units; the galactan moiety, which was also present, was predominantly found to be a backbone structure of (1→3) and (1→6) d-galactopyranose units with sulfation mostly on C-4 [15]. Later, a similar structure was found in FCSPs extracted from *Analipus japonicas* (Ectocarpales) [39].

The relatively large variations in the reported compositional and structural properties of the FCPSs extracted from different brown seaweed species thus clearly confirm the natural biodiversity of FCSPs notably as exemplified by the structures found in Fucales, e.g., in the *Fucus* sp. and *Sargassum* sp. (Figures 2 and 3). The (1→3)-linked α-l-fucopyranosyl backbone structure, with various extents of sulfate substitutions, is prevalent as the core backbone structure in the majority of the currently analyzed FCSPs. Nevertheless, the reported structural data for FCSPs from different brown seaweed species clearly indicate that there is no consistent basic structure of “fucoidan”. It also seems clear that FCSPs extracted from seaweeds under the same order have different composition, and in turn that the structural traits of FCSPs cannot be categorized or predicted according to algal order (Tables 1 and 2).

When assessing the available compositional data the large variation in the composition of the FCSPs products obtained from different extraction methods is evident (Table 2). Recently, we optimized the extraction yields of FCSPs from *Sargassum* sp. by developing a single-step extraction procedure. While doing so, we also systematically examined the effects of different extraction parameters (*i.e.*, acid concentration, time, and temperature) on the yields and composition of the FCSPs products obtained [19] (Table 2). All extraction factors affected the FCSPs yield. Lower total FCSPs yields, but higher fucose contents in the products were obtained with shorter extraction time [19]. The work also revealed that classical extraction treatment with HCl at elevated temperature and during extended time, *i.e.*, a procedure similar to the one used by Black *et al.* [9], had a detrimental effect on the FCSPs yield as this treatment apparently disrupted the structural integrity of the fucose-containing polymer and caused degradation of the chains built of fucose residues [19]. Hence, some of the classic methods, employing relatively harsh acid treatments, may in fact have affected the composition and structure of the target FCSP products to different extents, and may have contributed to the prevailing “conflicting views on the nature of fucoidin” recognized already in 1950 by Percival and Ross [8]. A consensus to employ defined extraction protocols for extraction of FCSPs, or at least an agreement among scientists in the field to include a benchmark extraction procedure in their studies, would help to advance the understanding of these intriguing FCSPs substances.

## 4. Bioactivity of Fucoidan or FCSPs

In recent years, fucoidan or FCSPs from seaweed biomass have been the subject of many scientific studies aiming at assessing their potential biological activities including antitumor and immunomodulatory [40–42], antivirus [43], antithrombotic and anticoagulant [44], anti-inflammatory [45], and antioxidant effects [46], as well as their effects against various renal [47], hepatic [48] and uropathic disorders [49].

Recently, low molecular weight FCSPs have been shown to have therapeutic potential in preventing intimal hyperplasia in both *in vivo* and *in vitro* studies: Contact with low molecular weight FCSPs (“fucoidan”) thus increased the migration of human vascular endothelial cells and induced decreased migration of vascular smooth muscle cells *in vitro* [50]. In an *in vivo* rat experiment FCSPs reduced the intimal hyperplasia in the rat aortic wall after balloon injury [50]. Furthermore, an *in vivo* efficacy study of fucoidan films conducted using a rat model showed that during cecal-sidewall surgery a fucoidan film wound healing treatment reduced the adhesion scores by approximately 90% and resulted in 50% to 100% of animals being adhesion free [51].

In this review, the most significant bioactivities of FCSPs, including antitumor and immunomodulatory, anticoagulant and antithrombotic effects will be presented with special focus on the relationship between the FCSP structural features and biological activity.

### 4.1. Antitumor and Immune-Response Activities

Several different therapeutic strategies such as chemotherapy, radiation therapy, surgery or combinations hereof have been used to treat different types of cancer. Unfortunately, several of these treatments provide only minimal benefits; moreover, there are undesirable complications and long term side effects of the treatments [52,53]. Consequently the quest for potential preventive or therapeutic measures against cancer has been going on for years and recently the focus has been directed towards bioactive compounds of natural origin, including FCSPs from brown seaweeds [1]. Many reports have been published which indicate the antitumor and immune-response modulating activity of FCSPs in both *in vivo* and *in vitro* studies [40–42,54,55].

Sulfated polysaccharide fractions from *Sargassum fulvellum*, *S. kjellmanianum*, *L. angustata*, *L. angustata* var. *longissima*, *L. japonica*, *Ecklonia cava*, and *Eisenia bicyclis* have been evaluated for their bioactivities, and they have been found to exert remarkable growth inhibitory activities on Sarcoma-180 cells implanted into mice and to possess antitumor activity against L-1210 leukemia in mice [56–58]. Recently, we reported the potent *in vitro* bioactivity of FCSPs extracted from *Sargassum* sp. and *F. vesiculosus* against lung and skin cancer cell growth [42]. The antitumor mechanism of FCSPs from sporophylls of *Undaria pinnatifida* has been described by Maruyama *et al.* [41,59]. The available findings indicate that antitumor activity of FCSPs may be associated with a significant enhancement of the cytolytic activity of natural killer (NK) cells augmented by increased production of macrophage-mediated immune response signaling molecules [59–61], namely interleukins (IL)-2, IFN-γ and IL-12 [42,59], and induction of apoptosis [42].

**Figure 5:**
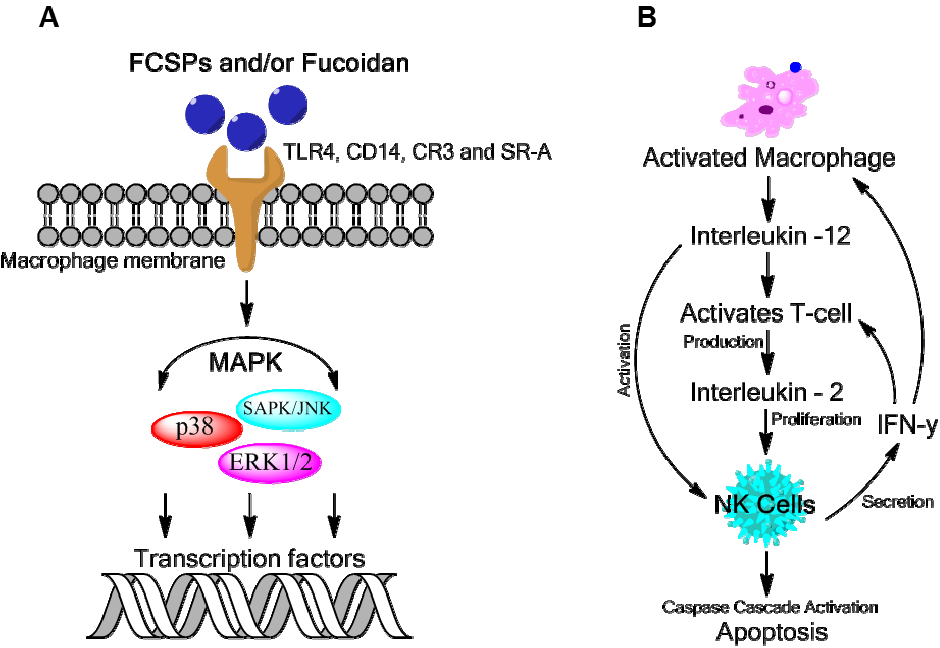
Proposed mechanism responsible for fucoidan bioactivity: (**A**) Macrophage activation by FCSPs as mediated through specific membrane receptor activation namely TLR-4, CD14, CR-3 and SR which in turn induce intracellular signaling via mitogen-activated protein kinases (MAPKs); (**B**) Activation of macrophages lead to production of cytokines such as IL-12, IL-2 and IFN-γ which enhance NK cell activation that may stimulate T-cell activation further via IFN-γ.

Macrophage activation by polysaccharides is mediated through specific membrane receptors. The major receptors reported for polysaccharides recognition in macrophages are glycoproteins including Toll-like receptor-4 (TLR-4), cluster of differentiation 14 (CD14), competent receptor-3 (CR-3) and scavenging receptor (SR) [61]. Activation of these receptors is mediated by intracellular signaling pathways and the family of mitogen-activated protein kinases (MAPKs) plays a critical role notably in the production of nitric oxide (NO) which can lyse tumors [61]. MAPK family members such as p38 MAPK, extracellular regulated kinase (EKR1/2) and stress-activated protein kinase/c-Jun-*N*-terminal kinase play an important role in the activation of macrophages by polysaccharides such as FCSPs [61,62] (Figure 5A). Activated MAPKs lead to activation of transcription factors resulting in induction of various genes [61]. Activation of macrophages induces the production of cytokines such as interleukin-12 (IL-12) which in turn stimulate the development of T-cells (Figure 5B). T-cells produce interleukin-2 (IL-2) that in turn activates NK cells proliferation. The NK cells themselves produce immunologically important cytokines, notably IFN-γ, which can further provoke the participation of macrophages in the stimulation of T-cell via induction of IL-12 [41,59] (Figure 5B).

NK cells appear to represent a first line of defense against the metastatic spread of blood-borne tumor cells, and normal NK activity may be important in immune surveillance against tumors [63]. NK-mediated killing of target cells by apoptosis is facilitated by activation of caspase cascades (Figure 5B). In tumor bearing mice, FCSPs appear to act as an immunopotentiator leading to increased antitumor effectiveness as exhibited by increased immune response against A20 leukemia cells and a lowering of the tumor size in transgenic (DO-11-10-Tg) mice [41]. Moreover, recent investigations of the immunomodulatory activity of FCSPs in rats with aspirin-induced gastric mucosal damage suggest that the gastro-protective effect of fucoidan against aspirin-induced ulceration may take place through the prevention of elevation of pro-inflammatory cytokines, IL-6 and IL-12 [64].

Apoptosis is one of the most prevalent pathways through which FCSPs can inhibit the overall growth of cancer. Previous studies have shown that different types of FCSPs can induce apoptosis in melanoma cells [42], HT-29 colon cancer cells [65], MCF-7 human breast cancer cells [66], and HS-Sultan human lymphoma cells [62]. In human HS-Sultan cells, the apoptosis may occur via activation of caspase-3 [62]; and in MCF-7 cells via caspase-8 dependent pathways [66]. Alternatively, FCSPs induced apoptosis may take place through activation of caspases via both death receptor-mediated and mitochondria-mediated apoptotic pathways [65].

### 4.2. Anticoagulant and Antithrombotic Activities

The earliest published report describing the anticoagulant activity of fucoidan was published in 1957 [67]. In that report it was shown that a certain fraction of fucoidan from *F. vesiculosus* possessed powerful anticoagulant activity that qualified fucoidan to belong to the group of heparinoids [67]. Heparin is a biomolecule containing highly sulfated glucosaminoglycan that is widely used as an injectable anticoagulant. It has been reported that the anticoagulant mechanisms of fucoidan are related to both antithrombin and heparin cofactor II-mediated activity [68,69], but the mechanisms by which fucoidan exerts anticoagulant activity remain controversial [32]. Hence, any possible relations between the physical and chemical properties, the structure, and the anticoagulant activity of fucoidan remain to be firmly established. The uncertainties are mainly due to the structural variation of fucoidan between algal species, but most likely also a result of the different extraction methodologies employed to isolate FCSPs that appear to have produced FCSPs of different composition, structure, and size, which have given rise to conflicting results in the detailed studies of mechanisms of anticoagulant activity [32,70].

Results obtained using the so called activated partial thromboplastin time assay (APTT) have strongly indicated that FCSPs from *F. vesiculosus* have specific anticoagulant activity. Comparable results have been obtained in two independent studies using FCSPs dosages equivalent to 9–13 U/mg *versus* 167 U/mg for heparin; and 16 U/mg *versus* 193 U/mg heparin, respectively [71,72]. When FCSPs samples isolated from nine brown seaweed species were tested for anticoagulant activities, the APTT results were significant at 12–38 U/mg as compared to at 167 U/mg for heparin [73]. A remarkable finding for anticoagulant action was also reported by Kitamura *et al.* [74], who showed that a FCSPs fraction from *L. angustata* var. *longissima* (Laminariales) had antithrombin activity at 200 U/mg, equivalent to a dose of 140 U/mg heparin. The particular FCSPs fraction having anticoagulant activity had a molecular weight of ~21–23 kDa and contained fucose-galactose-sulfate at a ratio of 9:1:9 with the sulfate substitutions at C-4 of the fucose residues [74].

It has been postulated that it is not a specific structural trait that determines fucoidan’s ability to elicit anticoagulant activity, but rather that the anticoagulant effect is due to a multitude of structural features including monosaccharide composition, molecular weight, sulfation level, and position of sulfate groups on the main chain of the polysaccharide [69,75–77]. The comprehensive study of the anticoagulant activity of fucoidan by Cumashi *et al.* [21] also noted that neither the content of fucose and sulfate nor other structural features affected the anticoagulant efficacy. FCSPs from *C. okamuranus* (Chordariales) have been reported to exert virtually no anticoagulant effect, and this could be due to the low amount of sulfate in its polymer backbone and/or the presence of vicinal branching points formed by 2-*O*-α-d-glucuronyl substituents (Figure 4) [21,14]. On the other hand, the concentrations of C-2 sulfate and C-2,3-disulfated sugar residues (Figure 2) have been reported to be a common structural feature for fucoidan anticoagulant activity [11]. The anticoagulant activity of high molecular weight FCSPs from *Ecklonia kurome* were thus reported to be dependent on both molecular weight and sulfate content [76]: Higher molecular weight FCSPs (*i.e.*, 27 and 58 kDa) showed higher anticoagulant activity than lower molecular weight FCSPs (*i.e.*, ≤10 kDa); and FCSPs samples having a high molar ratio of sulfate to total sugar residues were found to exhibit inhibitory effects on fibrinogen clotting by thrombin reaction [76]. These results were supported by data reported by Haroun-Bouhedja *et al.* [78] who reported a relationship between the extent of sulfate group substitutions and the biological activities of fucoidan. The anticoagulant activity of low molecular weight (LMW) fucoidan, *i.e.*, MW < 18 kDa was thus found to decrease with decreasing degree of sulfation, and very low-sulfate (<20%) or desulfated LMW fucoidan lost its anticoagulant activity, but retained some antiproliferative activity on CCL39 fibroblast cells [78]. In contrast, LMW fucoidan with sulfate content higher than 20% was found to exert profound anticoagulant activity as well as antiproliferative effects on fibroblast cell line CCL39 cells in a dose-dependent fashion [78].

Some studies suggest that also the sugar composition (e.g., fucose, galactose, mannose, etc.) or the type of oligo- or polysaccharides of the FCSPs may play an important role for anticoagulant activity [75,79]. However, the series of investigations conducted by Pereira *et al.* [32,80,81] indicated that a 2-sulfated, 3-linked α-l-galactan, but not α-l-fucan, was the potent thrombin inhibitor mediated by anti-thrombin of heparin cofactor II. These findings have however also pointed out that it is not necessarily the sugar composition but rather the sulfate substitutions on the sugars that determine the anticoagulant activity of fucoidan—or both [82].

Most of the reported studies were carried out with crude, diverse and complex FCSPs obtained via extraction from brown seaweeds as opposed to being chemically well defined structures. For this reason it is not easy to identify a structure *versus* activity relationship because of the presence of highly branched portions and the complex distributions of sulfate and acetyl groups in algal FCSPs. This aspect was attempted resolved by use of invertebrate polysaccharides [83]. The data obtained indicated that regular, linear sulfated α-l-fucans and sulfated α-l-galactans express anticoagulant activity, which is not simply a function of charge density, but critically dependent on the pattern of sulfation as well as monosaccharide composition. Sulfated α-l-fucans and fucosylated chondroitin sulfate were also shown to elicit antithrombotic activity when tested on *in vivo* models of venous and arterial thrombosis in experimental animals [83].

### 4.3. Bioactivities and Oversulfation of FCSPs

In 1984 crude FCSPs fractions from *Sargassum kjellmanianum* were prepared in order to investigate the influence of the sulfation levels on the survival of L-1210 leukemia bearing mice; and on the growth of Sarcoma-180 cells [58]. The study showed that the fraction with the highest sulfation was the most effective against L-1210 leukemia bearing mice and it produced an increase in life span of 26%. On the other hand, this particular FCSPs fraction was also less effective in inhibiting growth of Sarcoma-180 cells subcutaneously implanted into mice [58].

Since then, several investigations have focused on the effect of oversulfation of FCSPs on biological activity [54,78,84–89]. Oversulfated FCSPs may be obtained by further sulfation of native FCSP molecules using dimethylformamide as solvent and a sulfur trioxide-trimethylamine complex as the sulfating agent [86]. The inhibitory effects of such oversulfated FCSPs were investigated on the invasion of Murine Lewis Lung Carcinoma cells through a reconstituted membrane basement fragment, socalled laminin [86]. Oversulfated FCSPs were found to be the most potent inhibitor of tumor cell invasion and were also, in particular, found to inhibit tumor cell adhesion to laminin better than native and desulfated FCSPs. The most potent oversulfated FCSP structures had sulfate groups on both the C-3 and C-4 positions of the fucose units; hence, the particular spatial orientation of the negative charges in the FCSPs molecules may also be an important determinant of bioactivity [86]. The study did not allow firm conclusions to be drawn with respect to mechanisms of action, but it was suggested that the increased negative charge resulting from oversulfation might promote the formation of FCSPs-protein complexes involved in cell proliferation, in turn suppressing cell growth [86].

When the importance of the spatial orientation of the negative charges on the FCSPs was investigated in more depth it was confirmed that this feature plays a major role in determining the binding potency of FCSPs to vascular endothelial growth factor 165 (VEGF_165_) [88]. Both native and oversulfated FCSPs have been tested for their anti-angiogenic actions *in vivo* and for their *in vitro* anti-proliferative effects against B16 melanoma cells, Sarcoma-180 and Lewis lung carcinoma cells: The interaction of oversulfated FCSPs with VEGF_165_ occurred with high affinity and resulted in the formation of highly stable complexes, thereby interfering with the binding of VEGF_165_ to vascular endothelial growth factor receptor-2 (VEGFR-2). The results showed that both native and oversulfated FCSPs were able to suppress neovascularization in mice implanted Sarcoma-180 cells; and that both FCSPs types inhibited tumor growth through the prevention of tumor-induced angiogenesis, but the data indicated that sulfation tended to give more potent effects [88].

Native and oversulfated FCSPs derived from *Cladosiphon okamuranus* (Chordariales) were analyzed using ^1^H NMR spectroscopy and it was suggested that whereas natural sulfation produced 4-mono-*O*-sulfo-l-fucopyranose the oversulfated FCSPs contained 2,4-di-, 2-mono-, and 4-mono-*O*-sulfo-l-fucopyranose [89]. It was also suggested that sulfate content and the positioning of sulfate groups, e.g., 2,4-di- *vs.* 4-mono, might be important for the anti-proliferative activity of fucoidan in a human leukemia cell line (U937), an effect which is presumed to take place via induction of apoptosis associated with activation of caspase-3 and -7 [89].

The effects of oversulfation of low and high molecular weight FCSPs derivatives from *F. vesiculosus* and heparin on lipopolysaccharide (LPS)-induced release of plasminogen activator inhibitor-1 (PAI-1) from cultured human umbilical vein endothelial cells (HUVEC) were examined by Soeda *et al.* [87]. Their study demonstrated that all oversulfated FCSPs derivatives including high molecular weight derivatives of 100–130 kDa were effective in suppressing the LPS-induced PAI-1 antigen, and supported an important role of the degree of sulfation for bioactivity [87].

The correlation of oversulfation and conformation of molecular sizes of FCSPs for anticancer activity using human stomach cancer cell lines AGS was evaluated recently for FCSPs isolated from dried Undaria pinnatifada FCSPs [54]. The data showed that the oversulfated, low molecular weight FCSP derivatives increased the inhibition of cell growth, while the growth inhibition was less for native, high molecular weight FCSPs and for oversulfated high molecular weight FCSPs [54]. The differences were suggested to be a result of the smaller molecular weight fractions having a less compact conformation than the higher, which may have allowed a higher extent of sulfate substitution to occur during oversulfation.

## 5. Conclusions

Fucoidan—or FCSPs—are an important group of polysaccharides that show remarkable biological actions notably anticoagulant, antitumor and immune-response activities. Despite intensive research, the exact correlation between the bioactivity and the structural molecular features of FCSPs—which vary depending on seaweed species and extraction methodology—has yet to be clarified.

The preservation of the structural integrity of the FCSPs molecules nevertheless appears crucial for maintaining the biological properties and it has been clearly shown that the extraction treatment employed affects the composition and thus the structural features of the FCSPs substances.

The diverse structures and varied chemical composition of FCSPs may have hindered the development of an in-depth understanding of the precise properties of significance for specific bioactivity effects.

Important structural issues for bioactivity appear to include the degree of sulfation and the size of the FCSP molecules. Oversulfated FCSPs have thus been found to be potent inhibitors of tumor cell invasion compared to desulfated native FCSPs. Low molecular weight FCSPs have been shown to be effective in inhibiting human stomach cancer cell growth and to exert anticoagulant activity provided that the extent of the degree of sulfation was relatively high. Loss of anticoagulant activity has been observed with decreasing degree of sulfation, although anti-proliferative effects on fibroblast cell lines were retained.

Undoubtedly, the presence of impurities influences the biological properties of FCSPs and therefore may currently hinder our full understanding of the biological activity of fucoidan or FCSPs. Hence the development of standard extraction procedures for FCSPs including hydrolysis treatment, purification and fractionation methodology, preferably with specific steps adapted to the particular botanical order of the seaweed, will generate a better, common basis for analysis and understanding of bioactivities and the mechanisms determining the bioactivities of FCSPs. On this basis it may even be possible to target specific structural features and in turn tune the extraction procedure to obtain specific bioactivities via the use of targeted extraction methodologies.

Despite the availability of early, seminal studies of the extraction of FCSPs from brown seaweeds the understanding of the complex structures of FCSPs, is far from complete.

## Figures and Tables

**Figure 1:**
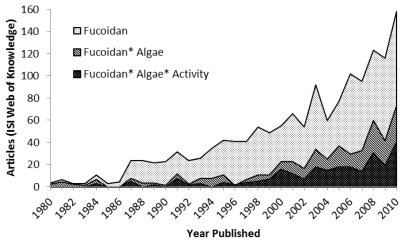
The trend during three decades of research on fucoidan as depicted by the number of published articles (Thomson Reuters, ISI Web of Knowledge). The number of articles was obtained according to topics being assigned in the ISI Web of Knowledge search engine with the following topic search terms: Fucoidan; Fucoidan*Algae; Fucoidan*Algae*Activity.

**Table 1:** Historic view of very early work, from 1913–1950, of fucoidan or FCSPs extraction and their reported composition from different brown seaweed species.

Year	Brown seaweed sp.	Reported FCSPs composition	Extraction method	References
1913	*Laminaria and Fucus*	Fucoidan contains fucose, that occurs together with mannitol, alginate and laminaran	Dilute acetic acid extraction	Kylin, 1913 [2]
1915	*Laminaria digitata*	Fucoidan contains l-fucose and other pentoses	Dilute acetic acid extraction	Kylin, 1915 [3]
1915	*Macrocystis pyrifera*	Mainly alginic acid, with some fucose-sulfate	Soaking in 2% Na_2_CO_3_ for 24 h, filtration, HCl addition, recovery of precipitate by filtration, redissolution in 2% Na_2_CO_3_	Hoagland and Lieb, 1915 [4]
1931	*Laminaria digitata*	Substantial amounts of calcium sulfate and uronic acid	Soaking of the seaweed in water, precipitation of crude, sulfated polysaccharides by ethanol	Bird and Haas, 1931 [5]
1931	*Macrocystis pyrifera*	Methylpentose monosulphate polymer with fucose and alginate contaminants	Repeated extraction with 2% HCl at room temperature for 48 h, precipitated with 90% ethanol	Nelson and Cretcher, 1931 [6]
1937	*Laminaria digitata*	Proposed (R-R′-O-SO_2_-OM)*_n_* with R = fucose, R′ as unknown, M being Na, K, Ca_0.5_, or Mg_0.5_	Precipitation of droplets exuded from seaweed in boiling ethanol	Lunde *et al.*, 1937 [7]
1950	*Fucus vesiculosus*, *Fucus spirales*, *Himanthalia lorea*, *Laminaria clustoni*	Substantial amounts of fucose and sulfate; small amounts of uronic acid, galactose and xylose; metals and ash were also detected, ash was mainly calcium sulfate	Aqueous extraction at ~100 °C for 24 h, extract treated with lead acetate (to precipitate alginate and proteins), filtrate solution treated with Ba(OH)_2_ to precipitate a “hydroxide-fucoidin complex”	Percival and Ross, 1950 [8]
1952	*Fucus vesiculosus*	Fucose, ash, sulfate	0.1 M HCl at pH 2–2.5 and 70 °C for 1 h, 3-times, fractional precipitation with ethanol	Black *et. al*., 1952 [9]

**Table 2:** Extraction methods and reported chemical composition of different brown seaweed species and their corresponding order.

Species	Order	Extraction method	Composition	Reference
*Cladosiphon okamuranus*	Chordariales	Seaweed-H_2_O suspension was treated with 30% HCl (pH 3) at 100 °C for 15 min. Supernatant was neutralized with NaOH, precipitated with CaCl_2_ and EtOH for 20 h at 4 °C, precipitate was dissolved with H_2_O then dried	fucose, glucose, uronic acid and sulfate	Nagaoka *et al.*, 1999 [14]
*Adenocystis utricularis*	Ectocapales	80% EtOH, 24 h, 70 °C pretreatment then extracted with water (or 2% CaCl_2_; or HCl) for 7 h at rt, followed by exhaustive extraction at 70 °C	fucose, rhamnose, glucose, galactose, xylose, mannose, uronic acid and sulfate	Ponce *et al.*, 2003 [15]
*Himanthalia lorea*	Fucales	Acid + alkali + water-acid-alkali sequence in 70 °C, 4 h.	fucose, xylose, uronic acid, sulfate	Mian and Percival, 1973 [12]
*Ascophyllum nodosum*	Fucales	Extracted at rt and then 70 °C with 0.01 NaCl containing 1% CaCl	fucose, xylose, galactose, glucose, sulfate	Marais and Joseleau, 2001 [16]
	Fucales	Extracted with hot water and dilute alkali, formaldehyde treatment, then extracted with ammonium oxalate-oxalic acid for 6 h at 80 °C	fucose, xylose, uronic acid sulfate	Percival, 1968 [17]
*Sargassum stenophyllum*	Fucales	Extracted with water 7% w/v mL, 12 h, 3×. Precipitated with EtOH and CaCl_2_ and cetylpyridinium chloride. Soluble fraction (SF) was then fractionated (F1–F6)	fucose, xylose, mannose, galactose, glucose, sulfate and uronic acid	Duarte *et al.*, 2001 [18]
*Sargassum* sp.	Fucales	Extracted with 0.03 M HCl at 90 °C for 4 h, single-step	Fucose, rhamnose, galactose, glucose, mannose, xylose, uronic acid, sulfate	Ale *et al.*, 2011 [19]
*Sargassum linifolium*	Fucales	Extracted with water at pH 1 (HCl), for 3 h at 80 °C	mannose, galactose, xylose, uronic acid and fucose residues	Abel-fattah *et al.*, 1974 [13]
*Fucus evanescens; Fucus distichus*	Fucales	Pretreatment: MeOH–CHCl_3_–H_2_O (4:2:1), then extracted 2% CaCl_2_ for 5 h at 85 °C, precipitated and the precipitate was washed with water, stirred with 20% ethanolic solution and dissolved with water [20]	fucose, xylose, galactose, uronic acid and sulfate	Cumashi *et al.*, 2007 [21]
*Fucus serratus*	Fucales	Pretreatment: MeOH–CHCl_3_–H_2_O (4:2:1), then extracted 2% CaCl_2_ for 5 h at 85 °C, the extracts were collected by centrifugation, combined, dialyzed and lyophilized [22]	fucose, xylose, mannose, glucose, galactose, uronic acid and sulfate	Cumashi *et al.*, 2007 [21]
*Hizikia fusiforme*	Fucales	Powdered seaweed was extracted with H_2_O (1:10), 3×, 2 h at 70 °C, precipitated with EtOH and CaCl_2_ then dried	fucose, mannose, galactose, xylose, glucose, rhamnose, arabinose, uronic acid and sulfate	Li *et al.*, 2006 [23]
*Laminaria saccharina; Laminaria digitata; F. vesiculosus; F. spiralis Ascophyllum nodosum*	Laminariales and Fucales	Extracted with 2% CaCl_2_ for 5 h at 85 °C, precipitated with Cetavlon, transformation of Cetavlonic salts into calcium salts, and an alkaline treatment to remove acetyl groups and to transform fucoidan into sodium salts [24]	fucose, xylose, mannose, glucose, galactose, uronic acid and sulfate	Cumashi *et al.*, 2007 [21]
*Chorda filum*	Laminariales	Extracted with CHCl_3_–MeOH–H_2_O (2:4:1) followed by 80% EtOH, then extracted successively with 2% CaCl_2_ at 20 and 70 °C, then with HCl (pH 2) and 3% Na_2_CO_3_, precipitated with calcium salt	fucose, xylose, mannose, glucose, galactose, uronic acid and sulfate	Chizhov *et al.*, 1999 [25]
*Undaria pinnatifida*	Laminariales	Ground seaweed extracted twice at rt for 6 h with 1% H_2_SO_4_, neutralized with 10% NaOH and lyophilized	fucose, mannose, xylose, rhamnose, galactose, glucose and sulfate	Hemmingson *et al.*, 2006 [26]
*Laminaria religiosa*	Laminariales	Water extraction at boiling temp. for 4 h, fucoidan fraction was obtained by using 0.09 HCl at 4 °C for 2 h, then precipitated with 85% EtOH and dried	fucose, xylose, mannose, glucose, rhamnose, uronic acid and sulfate	Maruyama and Yamamoto 1984 [27]
